# Ovarian Vein Thrombosis: Diagnosis and Management of an Unusual-Site Venous Thrombosis for the Acute Physician

**DOI:** 10.7759/cureus.117327

**Published:** 2026-10-01

**Authors:** Rebecca Walsh, Sibonokuhle Moyo, Sally Irwin

**Affiliations:** 1 Hematology, St Bartholomew's Hospital, London, GBR; 2 Geriatric Medicine, The Royal Free Hospital, Royal Free London NHS Foundation Trust, London, GBR

**Keywords:** anticoagulation, malignancy, ovarian, pregnancy, venous thromboembolism

## Abstract

Ovarian vein thrombosis (OVT) is a rare and under-recognised form of venous thromboembolism that is frequently missed due to absent or non-specific symptoms. Consequently, diagnosis is often delayed or made incidentally on cross-sectional imaging. Although OVT is usually associated with the postpartum period, it is increasingly recognised in patients with malignancy, pelvic surgery, and intra-abdominal infection. Potential complications may be life-threatening and include thrombus extension, pulmonary embolism, and, less commonly, sepsis and bleeding. Despite increasing recognition, evidence remains limited to small case series and reports. As such, there remains uncertainty regarding the optimal diagnostic approach and the use of anticoagulation, including indications for anticoagulation, duration of treatment, and follow-up. This review summarises the current evidence on the epidemiology, risk factors, clinical presentation, investigations, and management of OVT, and proposes a practical approach to the diagnosis and treatment of OVT for the acute physician.

## Introduction and background

Ovarian vein thrombosis (OVT) is a rare and under-recognised form of unusual-site venous thromboembolism that is frequently missed because of absent or non-specific clinical symptoms and signs, including abdominal pain, fever, nausea and vomiting, and sepsis [1,2]. Although typically associated with the postpartum period, OVT is increasingly recognised in patients with malignancy, recent pelvic surgery, and intra-abdominal infection [2]. Like other types of thrombosis, OVT risk factors are centred around Virchow’s triad, with venous stasis, hypercoagulability, and endothelial injury contributing to thrombus formation. Other risk factors may include prolonged immobility, thrombophilia, surgery, infection, pregnancy, hormone replacement therapy, and combined oral contraceptives [1,3]. The ovarian veins arise from the pelvic venous plexus, which surrounds the ovaries and fallopian tubes and ascends retroperitoneally along the psoas muscle. The left ovarian vein drains into the left renal vein, whilst the right drains directly into the inferior vena cava. A significant right-sided predominance has been reported in OVT, although left-sided and bilateral OVT can also occur. This may be due to anatomical and physiological differences associated with pregnancy, a significant known risk factor [2,4]. There is uncertainty regarding the optimum imaging modality for diagnosing OVT, with ultrasound, computed tomography (CT), and magnetic resonance imaging (MRI) being the main imaging modalities used [2]. Often, diagnosis is incidental during the work-up of other differential diagnoses or during malignancy staging or interval scans between cycles of chemotherapy or radiotherapy [2].

Delayed diagnosis may result in serious complications, including thrombus extension into the inferior vena cava, renal or iliac veins, or embolisation to the pulmonary circulation, and, less commonly, bleeding and sepsis [2,4]. The risk of complications may also be influenced by patient comorbidities, clinical status, underlying provoking factors, extent of thrombosis, and the presence of infection or malignancy [2,3,5]. As such, prompt clinical diagnosis and treatment are important to prevent further complications, which could lead to morbidity and mortality [1,3]. Despite increasing recognition, the evidence informing investigation and management remains limited; current practice is largely based on retrospective observational series, systematic reviews, and case reports, and large contemporary UK-specific datasets describing OVT appear to be lacking [2,5]. As a result, the clinical presentation, optimum management, and long-term outcomes and complications remain incompletely characterised [2,5].

The mainstay of treatment for OVT remains similar to that for other types of venous thrombosis, with anticoagulation [1,5]. However, the type of anticoagulant can vary, ranging from low-molecular-weight heparin to newer agents such as direct oral anticoagulants, but there is no consensus regarding the method or duration of anticoagulation [5]. There is uncertainty regarding the clinical significance and management of incidental, asymptomatic OVT, which has become increasingly common during malignancy staging and surveillance [5,6]. The recommended duration of treatment also appears to vary, usually within the range of 3-6 months, depending on underlying risk factors [5,7]. Improving understanding of OVT is essential to guide evidence-based diagnostic pathways and management guidelines, reduce morbidity, and improve patient outcomes. This review provides a practical overview of the epidemiology, risk factors, clinical presentation, diagnosis, and management of OVT, whilst highlighting areas of ongoing uncertainty and offering practical guidance for clinicians.

## Review

Methodology

This article is a narrative clinical review of the available literature on OVT, with a focus on its epidemiology, clinical presentation, risk factors, diagnosis, management, and clinical outcomes. A literature search was undertaken to identify relevant published evidence using the PubMed database. Additional relevant articles were identified by reviewing the reference lists of included studies. In total, 24 publications were identified. PubMed was selected as the sole database because it offered a comprehensive collection of clinical literature relevant to OVT, and sufficient studies were identified during the review process to inform the review. However, limiting the search to a single database may have resulted in relevant studies indexed elsewhere being missed. The search strategy combined terms relating to OVT and venous thromboembolism, including “ovarian vein thrombosis”, “puerperal ovarian vein thrombosis”, and related terms. A specific timeframe was not included in the search strategy. Articles were eligible for inclusion if they provided clinically relevant information regarding OVT, including observational studies, case series or reports, retrospective studies, and relevant systematic reviews. Studies were excluded if OVT was not the primary subject, if the article did not provide clinically relevant information, or if the full text was unavailable. There were no restrictions on study design, reflecting the limited and heterogeneous nature of the available evidence. No formal systematic review methodology or meta-analysis was undertaken. There was no formal assessment of study quality or risk of bias. 

Epidemiology and risk factors

OVT is a rare and under-recognised form of venous thromboembolism, classified as an unusual-site thrombosis due to its atypical anatomical location [1]. It is estimated to occur approximately 60 times less frequently than deep vein thrombosis (DVT), although the true incidence is likely underestimated [3]. OVT is associated with a broad range of risk factors, including malignancy, pelvic surgery, the postpartum period, and intra-abdominal or pelvic infections. Additional contributing factors may include a hypercoagulable state resulting from inherited or acquired thrombophilia. A literature review in 2024 involving 19 studies and 1,128 patients reported that the mean age at diagnosis was 37 years, and the frequency of OVT varied significantly depending on the clinical setting: malignancy was the most frequent association (37%), and OVT complicated 0.06% of deliveries, increasing to 0.19% following caesarean delivery [2]. However, most studies are observational and have low methodological quality; therefore, caution should be exercised when interpreting the findings.

The majority of published literature focuses on pregnancy-related OVT, which occurs in approximately 0.01%-0.18% of pregnancies [3]. In pregnancy- and postpartum-associated OVT, cases have been reported to be life-threatening in the absence of prompt treatment with antibiotics and anticoagulation [4]. A study comprising 219 patients with OVT found that 44% had an underlying malignancy, compared with 21% of patients with DVT [3]. Cancer was also strongly associated with mortality in the OVT cohort, highlighting the importance of considering underlying malignancy in non-pregnancy-associated OVT [3,5]. Yassa and Ryst reported OVT in 80% of patients following total abdominal hysterectomy (TAH); all cases were asymptomatic, with no CT evidence of phlebitis, and none of the patients had symptoms suggestive of pulmonary embolism (PE) [6]. The authors concluded that uncomplicated incidental OVT did not require treatment. OVT is rarely considered idiopathic, and cases of idiopathic OVT are not well-described in the literature. Idiopathic cases may highlight the need for more robust investigations, including consideration of underlying malignancy, infection screening, and thrombophilia testing, given the potentially high-risk nature of OVT. Case reports and small literature reviews of idiopathic OVT have described PE and have generally managed symptomatic cases according to the principles of conventional VTE treatment, including therapeutic anticoagulation [7]. The heterogeneity of these risk factors highlights the importance of maintaining a high index of clinical suspicion, especially in high-risk cohorts. 

Pathophysiology and anatomy 

The pathophysiology of OVT can be understood in the context of Virchow’s triad, which identifies three key factors that predispose to thrombosis: venous stasis, hypercoagulability, and endothelial injury. In OVT, the contribution of these risk factors depends on the underlying factors contributing to the thrombotic tendency. Venous stasis may be due to pelvic venous compression, local mass effect, or reduced mobility, which may be related to pregnancy or malignancy. Systemically, pregnancy causes increased levels of coagulation factors (I, VII, VIII, IX, X, XII, and von Willebrand factor) and reduced activity of natural anticoagulants (protein S, protein C, and antithrombin), contributing to the hypercoagulable state. Outside the pregnancy setting, malignancy is an important cause, with OVT associated with ovarian, cervical, endometrial, and other pelvic malignancies. Pelvic and systemic infections, such as endometritis, chorioamnionitis, and pelvic inflammatory disease, as well as malignancy or surgery involving the pelvis or abdomen, can lead to local inflammatory processes or vessel invasion, causing endothelial injury [2,8]. As such, in a young female with predisposing risk factors and persistent fever or abdominal pain despite appropriate antimicrobial therapy, OVT should be considered. Other risk factors include inherited or acquired thrombophilia, including factor V Leiden mutation, antiphospholipid syndrome, prothrombin mutation, and protein S or C deficiency. Monnet et al. reported thrombophilia in 18% of 201 patients who underwent thrombophilia testing; however, the indications for testing were generally not reported, and substantial selection bias limits interpretation of this finding [2]. Other less common causes of OVT include Crohn’s disease, inflammatory bowel disease, diverticulitis, and appendicitis [9].

The ovarian veins are retroperitoneal vessels that form from an extensive venous plexus surrounding the ovaries, fallopian tubes, and broad ligaments. The ovarian veins ascend retroperitoneally, generally along the psoas muscles, towards the renal vessels and inferior vena cava [10]. The pelvic venous system comprises multiple collateral pathways, which can be broadly classified into deep, superficial, and intermediate networks. Understanding these pathways may facilitate both diagnostic imaging interpretation and the planning of interventional procedures [11]. The right ovarian vein drains directly into the inferior vena cava just below the right renal vein, whereas the left ovarian vein drains into the left renal vein [12]. OVT demonstrates a marked right-sided predominance, with approximately 80% of cases affecting the right ovarian vein, 6% the left, and 14% being bilateral [13]. This distribution is thought to reflect anatomical and physiological differences, including the greater length of the right ovarian vein, antegrade blood flow, and less competent venous valves [12,14]. During pregnancy, dextrorotation of the enlarging uterus has been proposed as a contributing mechanism whereby compression of the right ovarian vein and inferior vena cava may promote venous stasis [2,15]. These anatomical relationships and patterns of venous drainage are particularly relevant when assessing thrombus extension, as right-sided OVT may propagate into the inferior vena cava, whereas left-sided thrombus may extend into the left renal vein or pulmonary circulation. These anatomical differences may also help explain the right-sided predominance of OVT.

Clinical presentation

OVT is frequently misdiagnosed or missed because symptoms may be absent or non-specific, such as abdominal pain, nausea and vomiting, fever, and gastrointestinal disturbance. Pelvic pain, abdominal mass, and fever have been described as a triad of symptoms associated with “septic pelvic thrombophlebitis” in OVT, which may be used as a guide for clinicians but should not be relied upon to exclude the diagnosis [5]. This diagnostic uncertainty can contribute to delays in diagnosis and potentially increase the risk of thrombus extension and other complications. These symptoms overlap with a range of more common intra-abdominal and pelvic pathologies, such as urinary tract infection, endometritis, appendicitis, pyelonephritis, surgical infections, or renal colic. In patients with malignancy, symptoms may be attributed to the underlying disease or chemotherapy- or radiotherapy-related complications. Similarly, in the postpartum period, OVT symptoms may mimic puerperal sepsis due to endometritis or retained products of conception. Hence, OVT may be identified incidentally on imaging performed for unrelated indications, such as malignancy staging, investigation of other differential diagnoses, or evaluation of postoperative complications. Patients with unexplained fever, tachycardia, or sepsis despite appropriate antibiotics should raise suspicion for OVT. On examination, abdominal tenderness, typically localised to the lower abdomen or flank and associated with guarding, may be present. Less commonly, a palpable abdominal mass may be identified. These findings are recognised clinical features of OVT. Furthermore, complications such as thrombus extension into the inferior vena cava or renal vein may produce more extensive venous symptoms, whilst PE may present with shortness of breath, hypoxia, chest pain, or haemodynamic instability. Similar to the clinical presentation of OVT, laboratory abnormalities such as elevated C-reactive protein, D-dimer, and leukocyte count may be present but are non-specific. A high index of clinical suspicion is therefore required, especially in patients with recognised risk factors who present with abdominal or pelvic pain, fever, or persistent symptoms despite treatment for an alternative diagnosis.

Diagnostic imaging

Diagnostic imaging plays a central role in the identification of OVT. The main modalities used to diagnose and evaluate OVT include pelvic MRI, ultrasonography, and contrast-enhanced CT, each offering distinct benefits and limitations in terms of diagnostic accuracy, accessibility, and cost. Doppler ultrasonography can identify a dilated ovarian vein containing intraluminal echogenic or hypoechoic thrombus with absent or reduced Doppler flow. This is an inexpensive and easily accessible modality that is commonly used in pregnant and postpartum patients [16]. However, it has lower diagnostic accuracy (sensitivity 50%-55.6%, specificity 41.2%-99%) and is limited by operator dependence, abdominal distension, overlying bowel gas, the deep retroperitoneal location of the vessels, and body habitus [2]. As such, a negative or inconclusive ultrasonography report cannot reliably rule out OVT, and when clinical suspicion of OVT remains high, cross-sectional imaging should be considered. Contrast-enhanced CT of the abdomen and pelvis is the most commonly used imaging modality in clinical practice due to its wide availability and ability to exclude alternative intra-abdominal pathology [17]. The characteristic finding of OVT is an enlarged ovarian vein containing a central low-attenuation filling defect, which may be associated with enhancement of the venous wall and surrounding inflammatory fat stranding. CT can demonstrate the full extent of the thrombus and its extension into surrounding structures, including the inferior vena cava, renal veins, or iliac veins. It can also evaluate alternative intra-abdominal or pelvic pathologies, including appendicitis, abscess, pelvic inflammatory disease, and pyelonephritis. These benefits must, however, be balanced against the risk of ionising radiation, particularly in young patients. Pelvic MRI appears to be an emerging gold standard, with the highest reported diagnostic accuracy, with a sensitivity of 92%-100% and specificity of 100% [2,18]. MRI provides excellent soft-tissue and vascular contrast, with OVT appearing as a filling defect within a dilated ovarian vein. This is particularly useful when CT or ultrasonography findings are inconclusive. Another advantage of MRI is that it does not expose patients to ionising radiation. However, in clinical practice, MRI use is frequently limited by cost, availability, and patient tolerance [2,18]. The choice of imaging modality should therefore be guided by a combination of clinical presentation, local availability, and the need to identify alternative pathology. A pragmatic approach for clinicians may be to use ultrasonography for the initial investigation, especially in pregnant and postpartum patients, with CT used when ultrasonography is inconclusive or when assessment of thrombus extension or alternative diagnoses is needed. MRI may be an alternative modality when CT is contraindicated, not possible, or inconclusive. CT and MRI remain the most valuable modalities for visualising the entire length of the ovarian vein and, crucially, identifying potentially life-threatening complications involving extension into the major abdominal veins [18,19].

Management**

The management of OVT remains challenging because of its rarity and the absence of randomised controlled trials specifically addressing treatment choices and OVT complications. Current recommendations are based on the application of general principles from other unusual-site venous thromboembolic conditions, observational studies, and expert opinion. Consequently, management is often individualised according to the underlying aetiology, symptom burden, thrombus extent, complications, and bleeding risk. Initial management and clinical assessment should determine whether there are any life-threatening features, including haemodynamic compromise, PE, or sepsis, and the patient should be stabilised accordingly. The presence of thrombus extension or PE strengthens the indication for therapeutic anticoagulation because of the high thrombotic burden. The subsequent management depends on the extent of thrombosis, provoking factors, and whether the patient is asymptomatic or symptomatic. OVT can generally be categorised into five groups, which differ in terms of management: postpartum state and pregnancy, surgery, malignancy, thrombophilia, and incidental OVT. OVT lies within the spectrum of venous thromboembolism, and the rationale for initiating anticoagulation is to prevent thrombus extension, PE, or recurrent venous thromboembolism whilst facilitating endogenous thrombus resolution.

Duration of anticoagulation and follow-up**

Anticoagulation is generally recommended for the treatment of symptomatic OVT, but there are no universally accepted OVT-specific guidelines. For non-pregnant patients without contraindications, treatment may be extrapolated from conventional deep vein thrombosis and PE management, where DOACs are generally preferred over vitamin K antagonists in appropriate patients [20]. However, the current literature on OVT does not establish which type of anticoagulant (low-molecular-weight heparin, warfarin, or direct oral anticoagulants) is preferable for treating OVT. For symptomatic postpartum OVT, Bannow and Skeith suggested anticoagulation for three months and antibiotics when infection is suspected, whilst recommending against anticoagulation for asymptomatic postpartum OVT unless there is thrombus extension or PE [17]. Low-molecular-weight heparin is generally favoured, particularly during pregnancy and breastfeeding, whilst the safety and efficacy of direct oral anticoagulants in breastfeeding women are not fully established [21]. However, for asymptomatic postpartum OVT, the routine use of anticoagulation is not recommended unless there is evidence of thrombus extension or PE [18]. The choice of anticoagulant in patients with active malignancy is more challenging and should take into account the tumour type, bleeding risk, tumour thrombus versus bland thrombus, ongoing cancer treatment, drug interactions, and renal function. In patients with malignancy, distinguishing incidental OVT from clinically significant thrombosis remains challenging, particularly when OVT is detected on surveillance imaging in asymptomatic patients. The appropriate duration of anticoagulation in this population is therefore not well-established [21]. Observational data from Riva et al. demonstrated low rates of major bleeding (1.27%) and recurrent venous thromboembolism (3.49%) among patients treated with heparin or vitamin K antagonists, supporting the safety and effectiveness of anticoagulation in appropriately selected patients [5]. Although anticoagulated patients had low rates of major bleeding and recurrent VTE, the observational evidence did not demonstrate a statistically significant reduction in recurrent VTE compared with non-anticoagulated patients. 

Further uncertainty remains regarding the benefit of anticoagulation for asymptomatic or incidentally detected OVT [17]. These cases may be identified following gynaecological or oncological surgery or during malignancy staging or interim scans. A retrospective study identified that women undergoing debulking surgery for ovarian cancer who developed incidental postoperative OVT rarely developed thrombus extension, and anticoagulation was generally not indicated, as clinically significant thrombus progression was uncommon [21]. These findings are consistent with the broader observational literature, suggesting that anticoagulation should not necessarily be given to every incidental, asymptomatic OVT, particularly when it occurs after pelvic cancer surgery without thrombus extension or PE [3,6,21]. In cases where thrombophilia is identified as the underlying driver of OVT, acute management remains the same, with therapeutic anticoagulation for symptomatic OVT. When thrombophilia is identified, anticoagulation decisions should incorporate the type of thrombophilia, whether the event was provoked, previous VTE history, and the ongoing risk of thrombosis. High-risk thrombophilia, recurrent VTE, or antiphospholipid syndrome may favour extended anticoagulation, whereas thrombophilia alone does not necessarily mandate indefinite treatment. The choice of secondary anticoagulation may favour an extended course of anticoagulation, and in high-risk antiphospholipid syndrome, anticoagulation with warfarin, a vitamin K antagonist, remains the preferred treatment [22]. When OVT occurs in the context of suspected pelvic infection or septic pelvic thrombophlebitis, antimicrobial therapy should be initiated alongside anticoagulation, with treatment tailored to the suspected source and microbiological findings [17]. Other measures, such as thrombolysis, thrombectomy, surgical thrombectomy, or inferior vena cava filter placement, are not routinely indicated for OVT. These measures should be reserved for exceptional circumstances and reviewed on an individual basis, such as life-threatening circumstances, an absolute contraindication to anticoagulation, or, rarely, progression or embolisation despite appropriate anticoagulation [17]. Historical techniques such as surgical thrombectomy or ligation are now rarely used. 

There is considerable variation in the duration of anticoagulation for OVT, with approximately 3-6 months being the most commonly reported treatment duration. The 2024 literature review by Monnet et al. identified low-molecular-weight heparin with or without subsequent oral anticoagulation for 3-6 months as the most frequently preferred management strategy [2]. Significant variation in anticoagulation duration was also reported by Riva et al.: 47.1% of patients were anticoagulated for 3 months or less, 35.3% for more than 3 months but no more than 6 months, and 17.6% for >6 months [5]. Pregnancy- and puerperium-associated OVT was often treated for a shorter duration, whereas longer treatment courses were more commonly used in malignancy-associated OVT. It would be reasonable to consider a shorter duration of anticoagulation in selected patients with a transient provoking factor, such as pregnancy or the postpartum period, provided that the patient is clinically improving and there is no persistent thrombotic risk, thrombus extension, or recurrent venous thromboembolism. However, patients with persistent provoking factors, such as active malignancy or thrombophilia, recurrent venous thromboembolism, or complications may be considered for a longer duration of anticoagulation. 

It remains controversial whether persistence of thrombus on follow-up imaging should lead to an extension of anticoagulation duration in OVT. Recanalisation is common but is not universal following appropriate anticoagulation for OVT. Riva et al. reported partial or complete ovarian vein recanalisation in 89.4% of anticoagulated patients but did not establish whether cases with persistent radiographic thrombosis were associated with recurrent venous thromboembolism or whether anticoagulation should be continued until complete recanalisation [5]. In a retrospective study of symptomatic OVT, 24% of patients had persistent occlusion despite anticoagulation, whilst 61% achieved complete recanalisation [23]. Persistent radiographic thrombus does not necessarily indicate treatment failure. Available observational studies have reported residual OVT despite anticoagulation, whilst the relationship between persistent thrombus and recurrent VTE remains uncertain [23,24]. Hence, routine serial imaging to document complete recanalisation is not clearly supported by current evidence and should be considered selectively. Repeat imaging may be appropriate when symptoms persist or worsen, when extension into the inferior vena cava or renal vein or PE is suspected, or when other complications develop. Therefore, the decision to continue or extend anticoagulation should be based primarily on the patient’s clinical course and underlying thrombotic risk rather than on radiographic persistence of thrombus alone.

The decision regarding anticoagulation duration should therefore be individualised according to the underlying thrombotic and bleeding risk factors, clinical response, thrombus extent, and risk of recurrent VTE, rather than based solely on a fixed treatment duration. In patients with a clearly transient provoking factor and an uncomplicated clinical course, discontinuation after approximately three months may be appropriate. In contrast, extended anticoagulation may be warranted when the provoking factor persists or when the risk of recurrent thrombosis is considered sufficiently high. Active malignancy represents a particular consideration, as the thrombotic risk may persist for as long as the malignancy remains active or systemic cancer treatment continues. Similarly, patients with recurrent venous thromboembolism or high-risk thrombophilia may require prolonged or indefinite anticoagulation, provided that the bleeding risk remains acceptable. Importantly, there is currently insufficient evidence to recommend extending anticoagulation solely until complete radiological resolution of the ovarian vein thrombus. Persistent thrombus on follow-up imaging may represent residual organised thrombus rather than ongoing acute thrombosis and does not necessarily indicate treatment failure. 

Complications 

OVT is generally associated with favourable clinical outcomes when recognised and treated promptly and appropriately. However, complications may arise due to thrombus extension, PE, infection and sepsis, and recurrent thrombosis. In the literature review by Monnet et al., involving 1,128 patients across 19 studies, thrombus extension to adjacent veins, including the iliac vein, inferior vena cava, and left renal vein, occurred in 5.9%, 10.3%, and 9.6% of patients, respectively. In OVT, the thrombus can propagate centrally and embolise to the pulmonary circulation. PE remains the most serious life-threatening complication and is particularly important when there is extension into the inferior vena cava or renal veins. In the literature review by Monnet et al., PE was reported in 6.5% of cases [2]. In some cases, inferior vena cava filters have been used prophylactically to reduce the risk of PE [25]. Septic puerperal OVT is associated with additional complications, including sepsis, septic thrombophlebitis, and bacteraemia, which can progress to septic shock and septic embolisation if left untreated. This supports the use of prompt and appropriate antimicrobial therapy alongside anticoagulation when infection is suspected [26]. Ovarian infarction and pelvic congestion syndrome are rarer complications but are clinically important because of their potential implications for ovarian function and, by extension, fertility. An enlarged thrombosed ovarian vein can encroach on and compress the adjacent ureter, leading to hydronephrosis or renal impairment. As with other types of venous thromboembolism, there is a risk of recurrent thrombosis, particularly when treatment is not initiated, anticoagulation is subtherapeutic, or the underlying thrombotic risk persists, including in the presence of malignancy and thrombophilia [9]. Across the included studies, recanalisation, recurrent thrombosis, and major bleeding were reported in approximately 70%, 8%, and 2% of patients, respectively [2]. It is important for clinicians to remain vigilant for OVT-related complications, given the potential for significant morbidity and mortality if these are not recognised and treated appropriately.

## Conclusions

OVT remains a rare, under-recognised, yet clinically important form of unusual-site venous thromboembolism. Its non-specific clinical presentation and frequent incidental detection can make diagnosis challenging, whilst the limited and heterogeneous evidence base creates uncertainty regarding optimal investigation and management. OVT is part of the spectrum of venous thromboembolism and should be considered in patients with non-specific symptoms, including fever persisting despite antibiotics, abdominal pain, and nausea and vomiting, particularly in the context of pregnancy, the postpartum period, malignancy, prothrombotic states, infection, or surgery. CT is widely used in clinical practice because of its availability, rapid acquisition, and ability to evaluate alternative intra-abdominal pathology, whereas MRI offers excellent diagnostic accuracy and may be particularly useful when CT findings are equivocal. Clinically important complications include thrombus extension into the inferior vena cava, renal or iliac veins, PE, and infection, underscoring the need for prompt recognition and appropriate treatment. Most patients are treated with anticoagulation, commonly low-molecular-weight heparin, for about 3-6 months; however, evidence on the optimal anticoagulant, treatment duration, and follow-up imaging remains limited. Management should therefore be individualised according to underlying provoking factors, thrombotic burden, bleeding risk, and clinical context. The limited evidence base and conflicting data regarding investigation and treatment make management more challenging than that of lower-limb DVT or pulmonary embolism. Larger prospective, multicentre UK studies are needed to better define the epidemiology and clinical characteristics of OVT and to inform evidence-based diagnostic and management guidelines.
